# Retaining individualities: the photodynamics of self-ordering porphyrin assemblies†
†Electronic supplementary information (ESI) available. See DOI: 10.1039/c5cc09095d



**DOI:** 10.1039/c5cc09095d

**Published:** 2015-12-18

**Authors:** Wen-Dong Quan, Anaïs Pitto-Barry, Lewis A. Baker, Eugen Stulz, Richard Napier, Rachel K. O'Reilly, Vasilios G. Stavros

**Affiliations:** a Department of Chemistry , University of Warwick , Gibbet Hill Road , Coventry , UK . Email: v.stavros@warwick.ac.uk ; Email: rachel.oreilly@warwick.ac.uk; b Molecular Organisation and Assembly of Cells Doctoral Training Center (MOAC DTC) , University of Warwick , Gibbet Hill Road , Coventry , UK; c School of Chemistry & Institute for Life Sciences , University of Southampton , Highfield , Southampton , UK; d School of Life Science , University of Warwick , Gibbet Hill Road , Coventry , UK

## Abstract

The retention of photochemical properties of individual chromophores is a key feature of biological light harvesting complexes. This is achieved despite extensive aggregation of the chromophores, which in synthetic chromophore assemblies often yields a change in spectral characteristics. As an alternative approach towards mimicking biological light harvesting complexes, we report the synthesis of porphyrin assemblies which retained the photochemical properties of the individual chromophore units despite their substantial aggregation. These new materials highlight a new bottom-up approach towards the design and understanding of more complex biomimetic and naturally occurring biological systems.

## Abstract 2

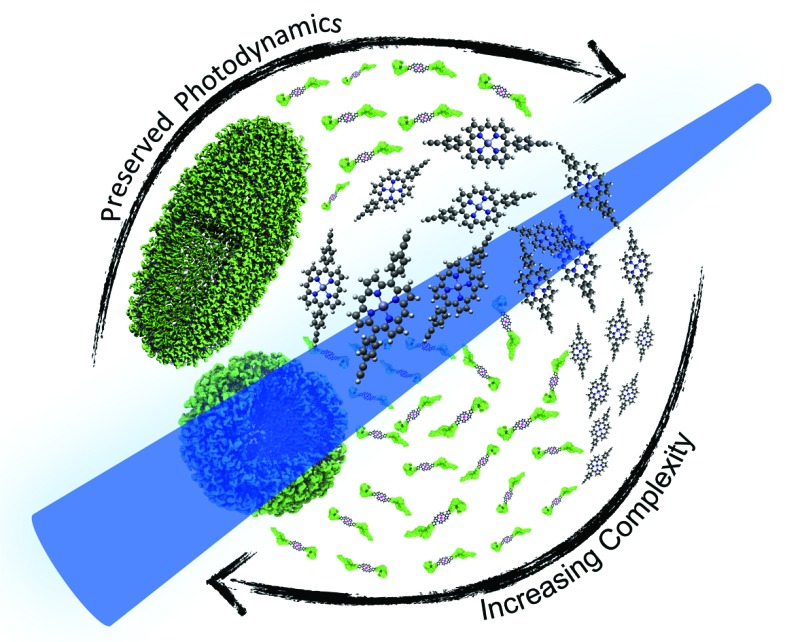
Retained photochemical properties – a simple porphyrin–polyDMA conjugate with the ability to self assemble into large (∼1 μm) vesicles in water. The photodynamics are remarkably preserved despite the extensive aggregation.

One of the most important processes for life on earth is photosynthesis, which is performed by plant and photosynthetic micro-organisms such as cyanobacteria. Nature uses light harvesting complexes (LHCs) to efficiently channel photoexcited energy on ultrafast time frames.^1–5^ This extraordinarily efficient process is enabled by the elegant and precise arrangement of chromophores,^3,6–8^ through sophisticated yet naturally-occurring self-assembly processes, and is an exemplar of evolution's phenomenal achievements. Natural LHCs have inspired numerous attempts to create synthetic mimics of such cyclic arrays of chromophores to enhance, for example, the efficiencies of photovoltaic cells.^9–11^ A template-directed synthetic method, elegantly demonstrated by the Anderson group, produced some of the closest mimics.^12–14^ These assembly processes and the requirement for covalent conjugation generally lead to altered spectral characteristics of the individual chromophores. Whilst this does extend the spectral coverage of single chromophoric systems, this is in contrast to biological LHCs, in which the UV-visible (UV-Vis) spectrum of the assembled system can usually be reconstructed by summing the UV-Vis spectra of the individual chromophores.^8,15,16^ Large aggregated and cross-linked systems are commonly utilised in biology for functions such as mechanical movements (actin filaments), photoprotection (melanin) and structural support (cross-linked cellulose and pectin), whereas the chromophores in LHCs are uniquely arranged through weak intermolecular interactions.^4,7,8^ Thus, the lack of covalent conjugations of these selected chromophores might be key to the functionalities of LHCs. Further, most of these synthetic assemblies require relatively high concentrations of the chromophores, and often challenging and complicated synthetic steps thus limiting their scalability and wider adoption.

In recent years, researchers have produced well-defined polymer-based self-assembled structures with relatively simple synthetic methods that are easily scaled up.^17–21^ In a handful of studies, various functionalised porphyrins, which are close mimics of some biological LHC chromophores, have been incorporated into these polymeric systems. These porphyrin–polymer conjugates were utilised in a range of applications such as photodynamic therapy (PDT),^22–24^ cell-imaging,^25,26^ initiators for complex polymers^27^ and simple proof-of-concept experiments for potential self-assembly methodologies.^28,29^ However, the only excited state dynamics studies performed involved long time scales (>nanoseconds), as the majority of the systems were oriented towards PDT applications.^30,31^ However, ultrafast dynamics of chromophores is a determinant of light energy harvesting efficiency.^1–3,32–37^ It is therefore crucial to understand the effects of such aggregation processes on the ultrafast photodynamics of individual chromophores to facilitate rational designs of LHC mimics based on non-covalent self-assembling polymers.

In an effort to address the aforementioned synthesis and assembly challenges, as well as to obtain further insight into their ultrafast excited state dynamics, we designed a simple proof-of-concept porphyrin–polymer conjugate (**Zn-dPP-pDMA**, Fig. 1a) to exploit the natural solvophobicity-driven self-assembly of amphiphilic systems. This was based on well-optimised *meso*-functionalised porphyrin synthesis methodologies^38–42^ and reported polymerisation methods.^43^ The careful selection of synthetic techniques produced **Zn-dPP-pDMA** in gram scale quantities. The relatively large scale synthesis, together with a lowered concentration for solvophobicity-induced assembly, produced polymer assemblies in quantities sufficient for condensed phase ultrafast transient electronic absorption spectroscopy (TEAS). Together with static photochemical studies, we demonstrate that the photochemical properties of individual chromophores are retained in these extensively aggregated systems. These experiments fill a gap in our knowledge, serving as an intermediate case study system that bridges the gap between the photochemical studies of simple small bio-molecules and complex macro-biological and biomimetic systems.

**Fig. 1:**
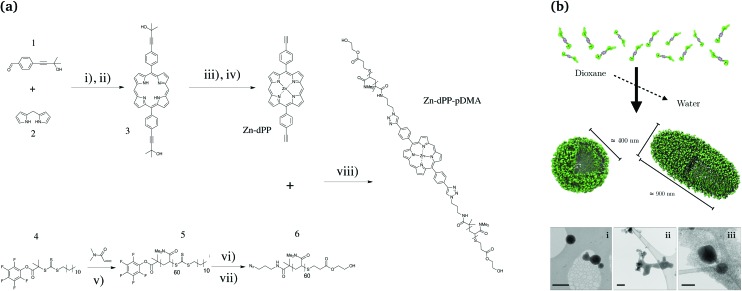
(a) Scheme for the synthesis of poly(dimethylacrylamide) functionalised Zn-porphyrin (**Zn-dPP-pDMA**). (b) Cartoon representation of the assembly, and their visualisation under cryo-TEM (bottom, i–iii, scale bars = 500 nm). Conditions in (a): (i) BF_3_·Et_2_O, CH_2_Cl_2_, N_2_, RT, 45 min; (ii) DDQ, toluene, N_2_, reflux, 3 h; (iii) Zn(OAc)_2_·(H_2_O)_2_, CH_2_Cl_2_ : MeOH (8 : 1), N_2_, 35 °C, 20 min; (iv) NaOMe, toluene, N_2_, reflux, 18 h; (v) AIBN, 1,4-dioxane, 65 °C, 40 min (80% conversion); (vi) 3-azidopropan-amine, tetrahydrofuran (THF), N_2_, RT, 18 h; (vii) HEA, PBu_3_, N_2_, THF, RT, 24 h; (viii) Cu·P(OEt)_3_, dimethylformamide, N_2_, RT, 48 h. Detailed procedures are provided in the ESI.†

The synthetic scheme for the preparation of **Zn-dPP-pDMA** is shown in Fig. 1a. All the synthetic techniques employed were based on readily optimised procedures,^38,39,41,43^ and resulted in respectable to quantitative yields (see ESI,† for further details). The azide-functionalisation and Z-group removal^44^ of the starting **pDMA** (**5**) was performed with an improved one-pot two-step aminolysis method.^45^ The conjugation of **6** and Zn-5,15-bis(4-ethynylphenyl)-porphyrin (**Zn-dPP**) *via* copper-catalysed azide alkyne cycloaddition was completed at room temperature within 48 h. The excess **pDMA** was easily removed by preparative size-exclusion chromatography (prep-SEC) in dioxane as the conjugated product is strongly coloured; dioxane was then removed effectively by lyophilisation. The resulting **Zn-dPP-pDMA** was assembled at 3 mg mL^–1^ (230 μM) by solvent switch from dioxane with slow addition of 18.2 MΩ cm water (see ESI†). Although we expected the formation of small micelles, cryogenic transmission electron microscopy (cryo-TEM) revealed surprisingly large vesicular polymersomes with spherical (Fig. 1b, i) and ellipsoid morphologies (Fig. 1b, iii). The irregular structures observed (Fig. 1b, i and ii) suggested that the assemblies were dynamic and undergoing both fusion and fission processes, similar to other reported polymer-based vesicles.^46^ Static/dynamic light scattering (SLS/DLS) characterisations at room temperature (RT, 20 °C) identified aggregates with *R*_g_ (radius of gyration) ≈ 470 nm and *R*_h_ (hydrodynamic radius) ≈ 190 nm (*R*_g_/*R*_h_ = 2.4), indicating that the majority of assemblies are ellipsoidal or undergoing the fusion/fission processes at RT.^47^ To verify that the large assemblies were indeed formed by the **Zn-dPP-pDMA**, we performed a series of characterisation experiments on samples filtered through membranes of different pore sizes. These studies showed that not only were large amounts of material remaining in the filter, the assemblies also underwent reorganisation, leading to significant change in size upon filtration (see ESI†). Thus, all photochemical experiments of these assemblies were performed with fresh, unfiltered samples, as shown in Fig. 1b.

Despite their extensive aggregation, the spectral features evidenced in the UV-Vis spectrum of **Zn-dPP** are largely retained in the assembled system (Fig. 2a). However, differences are apparent, which warrant discussion. Firstly, the Soret-band (≈ 414–420 nm, S_2_ ← S_0_) and Q-band (≈ 500–625 nm, S_1_ ← S_0_) are red-shifted by *ca.* 5 nm. Secondly, a broadening of the Soret band is evident. Lastly, there is an increase in Q-band relative to Soret-band intensities. These changes closely resemble that of a recently reported Mg(ii)bisporphyrin system, in which the Mg···Mg non-bonding distance was determined to be *ca.* 6.5–7.5 Å.^48^ These observations, taken together with the near identical fluorescence spectra of all the present systems (see ESI†) and the absence of excitonic features, as seen in reported dimers and ordered aggregates,^49,50^ described by Kasha's exciton theory,^51^ leads us to propose that while the chromophores are held at close proximity to each other, extensive stacking is prevented, with the chromophores of the aggregates being weakly coupled. This is very likely the result of the repulsive interactions between the polymer chains.^19,20^

**Fig. 2:**
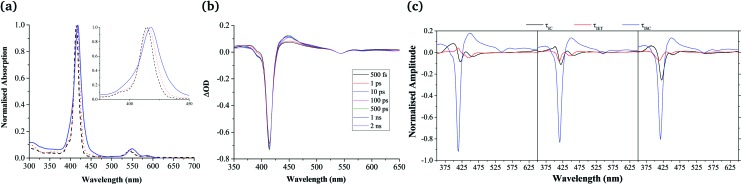
(a) Normalised UV-Vis spectra of **Zn-dPP** (black, dashed line), **Zn-dPP-pDMA** unimers in dioxane (red, dotted line) and **Zn-dPP-pDMA** assembled in water (blue, solid line). Inset shows zoomed-in Soret-band of each system. (b), TAS of **Zn-dPP** dissolved in dioxane photoexcited at 400 nm (2–5 mJ cm^–2^, 0.5 mm sample pathlength); ΔOD = change in optical density. (c) DAS of **Zn-dPP** (left), **Zn-dPP-pDMA** unimers (middle) fully solvated in dioxane and **Zn-dPP-pDMA** assembled in water (right). Amplitudes in (c) are normalised such that the sum of amplitudes at 416 nm equals to minus one.

After having established the general chromophore arrangement, we determined the excited state dynamics of the three systems (**Zn-dPP**, **Zn-dPP-pDMA** unimers solvated in dioxane; and **Zn-dPP-pDMA** assembled in 18.2 MΩ cm water), using TEAS following photoexcitation to the S_2_ state with 400 nm radiation. We first examined **Zn-dPP**, in which the transient absorption spectra (TAS) show two dominant features: the large ground state bleach (GSB) in the Soret region (*ca*. 416 nm) and the excited state absorption shoulders (ESA, *ca.* 450 nm, Fig. 2b). Global fitting the TAS^52–54^ reveals two ultrafast processes (Table 1): internal conversion (IC) of S_2_ → S_1_ (*τ*_IC_ ≈ 1 ps) and intermolecular vibrational energy transfer (IET) between the **Zn-dPP** S_1_ excited state and the dioxane solvent bath (*τ*_IET_ ≈ 21.8 ps). These time constants and corresponding processes are comparable to the previously studied model Zn-tetraphenyl-porphyrin (**Zn-tPP**) reported by Zewail and co-workers (see ESI†).^55^ We note that in addition to these two extracted time-constants, there is a time-constant that extends beyond the temporal window of our measurements (2 ns) which, in accord with previous studies,^55^ we attribute to intersystem crossing (ISC) of S_1_ → T_*n*_ (*τ*_ISC_) (see Table 1, footnote *a*). The shapes of the decay associated spectra (DAS, Fig. 2c) are highly informative in guiding the interpretation of the TAS. In particular, any negative components correspond to an exponential rise in that population whilst any positive components correspond to an exponential decay in population. The flow of excited state populations can be visualised in the DAS when a positive and negative features appear concomitantly. This can be interpreted as either a change in electronic state (IC or ISC), or relaxation within a single electronically excited state (IET).^52,56^

Remarkably, almost identical features are observed in the TAS of the functionalised systems (**Zn-dPP-pDMA** unimers in dioxane and assembled in water, see ESI†). Furthermore, these are fitted with almost identical time constants (Table 1) and the DAS (Fig. 2c) revealed no discernible differences in their features. Of these, *τ*_IC_ showed insignificant variations. The slightly faster *τ*_IET_ observed in the assembled system (15.2 ps *cf.* 20.3 ps in dioxane) suggests that the vibrational frequency match between the Franck–Condon active modes of the photoexcited **Zn-tPP** and the instantaneous normal modes of its surrounding molecules might be different between each system, as inferred in previous studies.^55,57^ However, the differences are within the 95% confidence interval of each other (Table 1 and ESI†), which makes this supposition tentative. The final ISC process demonstrated no discernible difference within the window of our experiments (Table 1 and Fig. 2c).

**Table 1:** Global fitted time constants of each system studied (*τ*_*n*_)^*a*^

System studied	*τ*_IC_	*τ*_IET_	*τ*_ISC_
**Zn-dPP** dioxane	1.0 ± 0.3 ps	21.8 ± 8 ps	≫2 ns
**Zn-dPP-pDMA** unimers in dioxane	1.0 ± 0.3 ps	20.3 ± 8 ps	≫2 ns
**Zn-dPP-pDMA** assembled in water	0.8 ± 0.3 ps	15.2 ± 6 ps	≫2 ns

^*a*^Due to the very large signal intensities attained at time zero (likely multicomponent in nature and attributed to linear and non-linear solvent-, glass-, and solute-only responses), which extend to ∼150 fs, this signal was excluded from the global fits.

In conclusion, we have presented a study on a basic system of solvated and aggregated porphyrin molecules assembled *via* solvophobicity. The photodynamic studies presented demonstrate that the individual **Zn-dPP** molecules retained their overall photochemical properties following the addition of a large polymer chain (pDMA), even following assemblies into macromolecular vesicles. The fact that the addition of such a large polymer has very little effect on the photochemical properties of the porphyrin adds credence to the ‘bottom-up’ approach towards understanding the photochemistry and photophysics of complex biological systems.^58–61^ Coupled with the relatively high yielding synthetic steps and simple assembly method, these types of polymer–chromophore conjugates could be opportune building blocks for more complex biomimetic systems. We propose that this proof of concept study should facilitate future modular designs of photo-active biomimetic arrays which do not rely on the complex covalent conjugation of multiple chromophores, thereby allowing full exploitation of individual pigment characteristics.

W.D.Q. thanks Dr A. M. Sanchez, Mr Ian Hands-Portman and Miss L. J. MacDougall (UoW) for their help and discussions on TEM and SEM instruments. The research leading to these results has received funding from the ERC under the EU 7th Framework Programme/ERC grant no. SCPs 615142; the EPSRC equipment grant EP/J007153; EPSRC studentship grant EP/F500378/1; and the RSURF scheme.
