# Supplementary material for: Retaining individualities: the photodynamics of self-ordering porphyrin assemblies
Source: Chem Commun (Camb). 2015 Dec 18;52(9):1938–41. doi: 10.1039/c5cc09095d (PMC4720934; doi:10.1039/c5cc09095d)
Supplement: Supplementary file 1 [file CC-052-C5CC09095D-s001.pdf]

Electronic Supplementary Material (ESI) for ChemComm.
This journal is © The Royal Society of Chemistry 2015

Electronic Supplementary Information

for

Retaining Individualities: the Photodynamics of Self-Ordering Porphyrin Assemblies

Wen-Dong Quan[a,b], *Anaïs Pitto-Barry*[a], *Lewis A. Baker*[a,b], *Eugen Stulz*[c], *Richard Napier*[d], *Rachel K. O'Reilly*[a*] and Vasilios G. Stavros[a*]

[a]*Department of Chemistry, University of Warwick, Gibbet Hill Road, Coventry, UK.*
E-mail: v.stavros@warwick.ac.uk; r.k.o-reilly@warwick.ac.uk
[b]*Molecular Organisation and Assembly of Cells Doctoral Training Center (MOAC DTC),University of Warwick, Gibbet Hill Road, Coventry, UK.*
[c]*School of Chemistry & Institute for Life Sciences, University of Southampton, Highfield, Southampton, UK.*
[d]*School of Life Science, University of Warwick, Gibbet Hill Road, Coventry, UK.*

Contents

1 Experimental **1**

1.1 Materials . . . 2

1.2 Apparatus . . . 2

Time Resolved Transient Electronic Absorption Spectroscopy (TEAS) . . . 3

1.3 Synthesis Procedures . . . 4

4,4'-(porphyrin-5,15-diylbis(4,1-phenylene))bis(2-methylbut-3-yn-2-ol) (**3**) . . . 4

Zn-4,4'-(porphyrin-5,15-diylbis(4,1-phenylene))bis(2-methylbut-3-yn-2-ol (**3a**) . . . 4

Zn-5,15-bis(4-ethynylphenyl)-porphyrin (**Zn-dPP**) . . . 5

Azido-amine-poly(DMA)$_{60}$-thiol-HEA (**6**) . . . 6

Zn-5,15-bis(4-(1-poly(DMA)$_{60}$-1H-1,2,3-triazole-4-yl)phenyl)-porphyrin (Zn-dPP) (**Zn-dPP-pDMA**) . . . 7

1.4 Infra-Red (IR) Spectra . . . 8

1.5 Size Exclusion Chromatography . . . 9

1.6 Assembly of Zn-dPP-pDMA . . . 9

2 Morphological Characterisation of Assembled Systems **10**

2.1 Light Scattering . . . 10

2.2 UV-Visible Absorption of Filtrated Samples . . . 11

2.3 Transmission Electron Microscropy Studies . . . 12

3 Photochemical Information **13**

3.1 Static Fluorescence . . . 13

3.2 Time-resolved Transient Electronic Absorption Spectroscopy (TEAS) . . . 15

Global Fitting and Error Analysis . . . 15

Transient Absorption Spectra (TAS) . . . 17

Lifetime Uncertainties . . . 18

1 Experimental

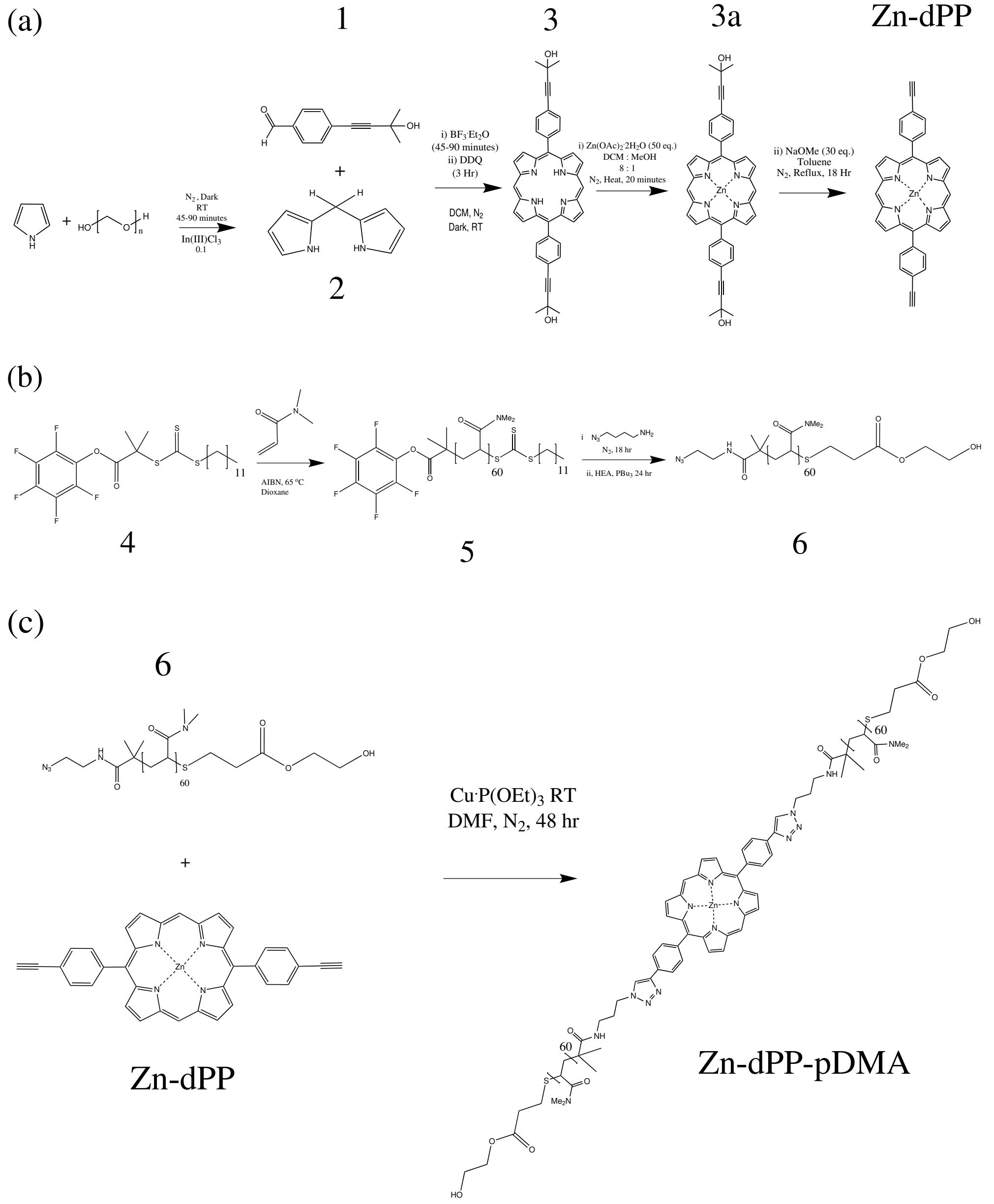

Scheme S1 Overall synthesis scheme for (a) 5,15-bis(4-ethynylphenyl) porphyrin (**Zn-dPP**); (b) Azide-functionalised poly(dimethylacrylamide) (**6**); (c) Zn-5,15-bis(4-(1-poly(DMA)$_{60}$-1H-1,2,3-triazole-4-yl)phenyl)-porphyrin (**Zn-dPP-pDMA**) by copper-catalysed azide alkyne cycloaddition of **6** to **Zn-dPP**.

1.1 Materials

Spectroscopic grade 1,4-dioxane was purchased from VWR. HPLC grade solvents were purchased from Fisher. Water for synthesis, spectroscopy and self assembly was purified to a resistivity of 18.2 MΩ·cm using a Millipore Simplicity Ultrapure water system. 4-(3-hydroxy-3-methylbut-1-ynyl) benzaldehyde (**1**) was synthesised according to procedures by Stulz and coworkers.[1] *S*-Dodecyl-*S*'-(α,α-dimethylpentafluorophenyl acetate)trithiocarbonate chain transfer agent (CTA, **4**) was synthesised according to procedures by Godula *et al.*[2] Dipyrrolemethane (**2**) was synthesised according to procedure described by Laha *et al.*[3] Reversible Addition-Fragmentation chain Transfer (RAFT) polymerisation of dimethylacrylamide (**5**, DP $\approx$ 60) with CTA **4** was performed as previously reported.[4] Pyrrole was purchased from Aldrich and distilled over CaH_2 under vacuum (0.5 mBar) and stored under N_2 protected from light prior to use. All other chemicals and solvents were purchased from Sigma, Aldrich, Fluka or Acros and used as received unless stated otherwise. Dialysis was performed using Spectra/Por® of appropriate molecular weight cut off (MWCO), purchased from VWR or Fisher. Preparatory size exclusion chromatography (prep-SEC) was performed with Bio-Beads™ S-X Resin in appropriate solvents, purchased from Bio-Rad. All dry-state transmission electron microscope (TEM) samples were prepared on graphene oxide (GO)-coated carbon grids (Quantifoil R2/2).[5] Generally, a drop of sample (20 µL) was pipetted on a grid, blotted immediately and left to air dry. For cryogenic electron microscopy (cryo-TEM), the samples were prepared at ambient temperature by placing a droplet on a TEM grid. The extra liquid was then blotted with a filter paper and the grid was inserted into liquid ethane at its freezing point. The frozen samples were subsequently kept under liquid nitrogen.

1.2 Apparatus

^{1}H and ^{13}C NMR spectra were recorded on a Bruker DPX-300, DPX-400, or AV-250 spectrometer at room temperature. Chemical shifts are given in ppm downfield from the internal standard tetramethysilane (TMS). Infrared spectra were recorded on a Perkin Elma, spectrum 100 FT-IR spectrometer. Fluoresence spectra were recorded using an Agilent Cary Eclipse Fluorescence spectrophotometer. High resolution mass spectrometry (HR-MS) was conducted on a Bruker UHR-Q-ToF MaXis with electrospray ionisation. UV-Vis

spectroscopy was carried out on a Perkin Elma Lambda 35 UV/Vis spectrometer. Quartz cells with screw caps and four polished sides (Starna) were used for fluorescence and UV-Vis measurements. The specific refractive index increment (dn/dc) of the polymers were measured on a refractometer (Bischoff RI detector) operating at $\lambda_0 = 632$ nm. Laser light scattering measurements were performed at angles of observation ranging from 15° up to 150° with an ALV CGS3 setup operating at $\lambda_0 = 632$ nm and at 20 °C. Data were collected in duplicate with 150-300 s run times. Calibration was achieved with filtered toluene and the background was measured with filtered 18.2 MΩ·cm water. TEM observations were performed on a JEOL 2000FX electron microscope at an acceleration voltage of 200 kV. Cryo-TEM was performed with a JEOL 2010F TEM, operated at 200 kV with images recorded on a Gatan UltraScan 4000 camera.

Time Resolved Transient Electronic Absorption Spectroscopy (TEAS)

The detailed experimental procedures for TEAS can be found in previous reports.[6–8] Briefly, a commercially available Ti:sapphire oscillator and amplifier system (Spectra-Physics) produces 3 mJ laser pulses of ≈ 40 fs duration centered around 800 nm with a repetition rate of 1 kHz. For TEAS, a 1 mJ/pulse 800 nm laser beam is split into two beams of (i) 0.95 and (ii) 0.05 mJ/pulse. Beam (i) is used to generate the pump pulse centered around 400 nm (2-5 mJ·cm^{-2}) through second harmonic generation using a β-barium borate crystal. Beam (ii) is used to generate the probe pulse, a white light continuum (330-725 nm). Pump-probe polarizations are held at the magic angle (54.7°) relative to one another. Changes in optical density (ΔOD) of the sample are calculated from probe intensities, collected using a spectrometer (Avantes, AvaSpec-ULS1650F). The delivery system for the samples is a flow-through cell (Demountable Liquid Cell by Harrick Scientific Products, Inc.). The sample is circulated using a PTFE tubing peristaltic pump (Masterflex), recirculating sample from a 50 mL reservoir, in order to provide each pump-probe pulse pair with fresh sample.

1.3 Synthesis Procedures

4,4'-(porphyrin-5,15-diylbis(4,1-phenylene))bis(2-methylbut-3-yn-2-ol) (3)

Compound **1** (1.411 g, 7.5 mmol, 1 eq.) and **2** (1.096 g, 7.5 mmol, 1 eq.) were dissolved in 750 mL of CH_2Cl_2. The solution was purged with dry N_2 for 45 minutes before adding $BF_3 \cdot Et_2O$ (600 µL, 4.5 mmol, 0.6 eq.). The solution was protected from light. After stirring the solution for 45 minutes at RT, 2,3-dichloro-5,6-dicyano-1,4-benzoquinone (DDQ, 1.532 g, 6.75 mmol, 0.9 eq.) was added to the reaction. The reaction was stirred for one hour and the crude mixture was filtered through a neutral aluminium oxide patch (7.5 cm) and washed with 5% methanol in CH_2Cl_2 until the eluent was colourless. The crude product was dried *in vacuo* and re-dissolved in 150 mL of toluene. A fresh batch of DDQ (1.702 g, 7.5 mmol, 1 eq.) was added and the mixture was heated to reflux for 3 hrs. The crude reaction was passed through a neutral aluminium oxide patch again before being purified by flash chromatography (silica, neutralised with 1% triethylamine, 5% ethylacetate in $CH_2Cl_2 \rightarrow$10% EtOAc in CH_2Cl_2), yielding purple solid products (334 mg, 0.53 mmol, 16%) ^{1}H NMR ($CDCl_3$, 400 MHz, ppm) δ = 1.79 (s, 12H), 2.05 (br, 2H), 7.87 (d, $^3J_{\text{H-H}}$ = 8 Hz, 4H), 8.21 (d, $^3J_{\text{H-H}}$ = 8 Hz, 4H), 9.05 (d, $^3J_{\text{H-H}}$ = 5 Hz, 4H), 9.40 (d, $^3J_{\text{H-H}}$ = 5 Hz, 4H), 10.32 (s, 2H); ^{13}C NMR ($CDCl_3$, 400 MHz, ppm) δ = 31.4, 130.3, 130.3, 131.9, 134.8; HR-MS (MaXis) m/z found 627.2733, calc. 627.2755 ($[C_{42}H_{34}N_4O_2+H]^+$, 100%).

Zn-4,4'-(porphyrin-5,15-diylbis(4,1-phenylene))bis(2-methylbut-3-yn-2-ol (3a)

Zn(II) acetate dihydrate (1.756 g, 8 mmol, 50 eq.) and compound **3** (10 mg, 16 µmol, 1 eq.) were dissolved in 9 mL methanol/DCM (1 : 8), degassed with N_2 for 20 minutes. The solution was then heated to 35 °C for 20 minutes. The mixture is then dried *in vacuo* and dissolved in 2 mL CH_2Cl_2, filtered to remove excess Zn(II) acetate dihydrate. Solvent was removed under reduced pressure, affording pinkish-purple solid products (10.4 mg, 14.4 µmol, 94%) ^{1}H NMR ($CDCl_3$, 400 MHz, ppm) δ = 1.79 (s, 12H), 7.87 (d, $^3J_{\text{H-H}}$ = 8 Hz, 4H), 8.21 (d, $^3J_{\text{H-H}}$ = 8 Hz, 4H), 9.05 (d, $^3J_{\text{H-H}}$ = 4 Hz, 4H), 9.41 (d, $^3J_{\text{H-H}}$ = 4 Hz, 4H), 10.34 (s, 2H); ^{13}C NMR ($CDCl_3$, 400 MHz, ppm) δ = 33.6, 129.2, 129.8, 131.7, 134.7; HR-MS (MaXis) m/z found 711.1694, calc. 711.1709 ($[C_{42}H_{32}N_4O_2+Na]^+$, 100%).

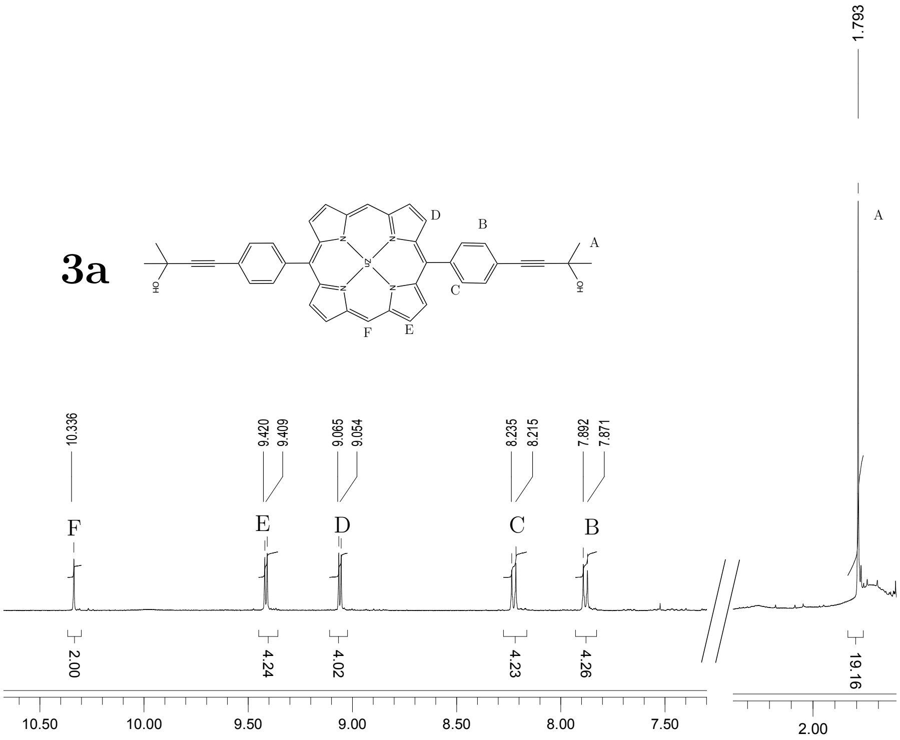

Figure S1 ^{1}H NMR spectra of **3a** in $CDCl_3$

Zn-5,15-bis(4-ethynylphenyl)-porphyrin (Zn-dPP)

NaOMe (24 mg, 432 mmol, 30 eq.) and compound **3a** (10.4 mg, 14.4 μmol, 1 eq.) were dissolved in 15 mL of toluene, degassed with N_2 for 20 minutes before heating to reflux (125 °C). The reaction was left over night. Solvent was removed *in vacuo*, crude product was extracted with 20 mL DCM, washed with water (3 × 100 mL) then brine (2 × 100 mL) and dried with $MgSO_4$. Basic alumina was added to the filtered solution, dried *in vacuo* before loading onto the column (neutralised silica, eluent: $CH_2Cl_2 \rightarrow$ 0.5% Methanol in CH_2Cl_2). This afforded pinkish-purple solid products (7.4 mg, 13.0 μmol, 90%). ^{1}H NMR ($CDCl_3$, 400 MHz, ppm) δ = 3.35 (s, 2H), 7.93 (d, $^3J_{\text{H-H}}$ = 8 Hz, 4H), 8.23 (d, $^3J_{\text{H-H}}$ =8 Hz, 4H), 9.12 (d, $^3J_{\text{H-H}}$ = 5 Hz, 4H), 9.46 (d, $^3J_{\text{H-H}}$ = 4 Hz, 4H), 10.36 (s, 2 H); ^{13}C NMR ($CDCl_3$, 400 MHz, ppm) δ = 130.5, 132.1, 132.3, 134.6.

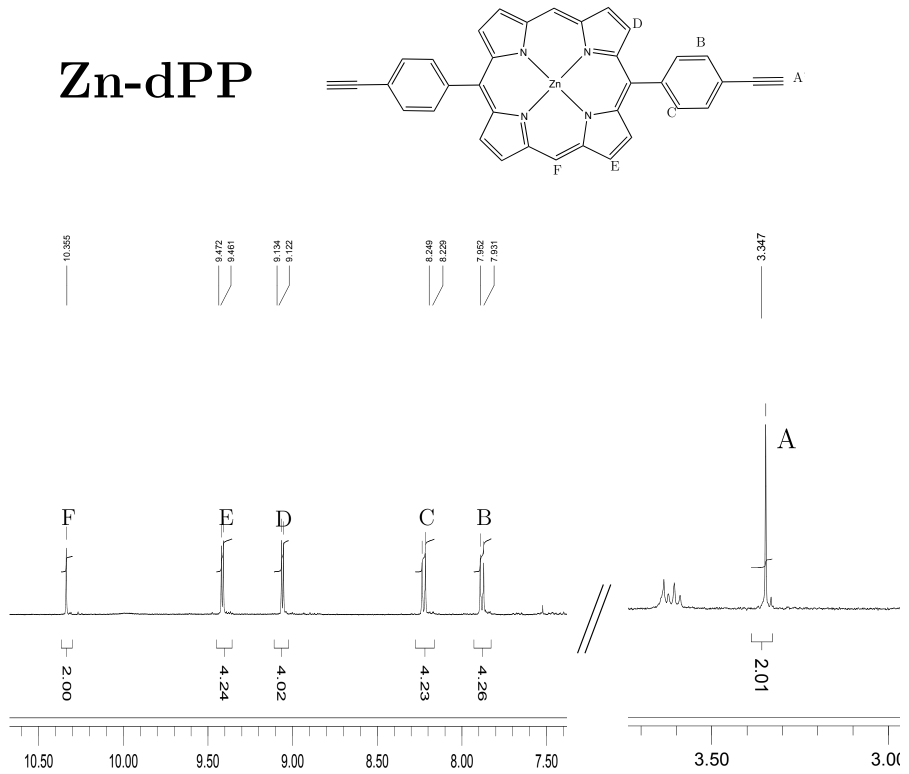

Figure S2 ^{1}H NMR of **Zn-dPP** in $CDCl_3$.

Azido-amine-poly(DMA)$_{60}$-thiol-HEA (6)

Polymer **5** (1.5 g, 0.25 mmol, 1 eq.) and 3-azidopropan-amine (75 mg, 0.75 mmol, 5 eq.) were dissolved in THF, 30 mL and 1 mL, respectively, in separate oven-dried ampoules and degassed by three freeze-pump-thaw (FPT) cycles. The 2-azidopropan-1-amine solution was then added to the polymer solution *via* cannular transfer under dry N_2. The solution turned from dark yellow in colour to a clear-pale yellow-green colour after being stirred at RT for 24 hrs. Neat 2-hydroxyethyl acrylate (2.9 g, 25 mmol, 100 eq.) and PBu_3 (1.011 g, 5 mmol, 20 eq.) were placed in separate oven-dried ampoules and degassed by three FPT cycles and added to the clear reaction solution *via* cannula transfer under dry N_2. The final mixture was stirred at RT for 24 hrs. 100 mL of 18.2 MΩ·cm water was added to the mixture, turning the solution from pale yellow to a cloudy-white colour. The resulting solution was dialysed against water using dialysis tubing with 3.5k MWCO over a minimum of 6 water changes to remove excess starting material. The water was

removed by lyophilisation, recovering the polymer in quantitative yield.

Zn-5,15-bis(4-(1-poly(DMA)$_{60}$-1H-1,2,3-triazole-4-yl)phenyl)-porphyrin (Zn-dPP) (Zn-dPP-pDMA)

Polymer **6** (800 mg, 133 µmol, 2.5 eq.), **Zn-dPP** (30.4 mg, 66.7 µmol, 1 eq.) and Cu·P(OEt)$_3$ (48 mg, 133 µmol, 2.5 eq.) were dissolved in 15 mL of DMF. The solution was purged with dry N_2 for 30 minutes then stirred under N_2 at RT for 48 hrs. 50 mL of 18.2 MΩ·cm water was then added to the reaction and dialysed against 18.2 MΩ·cm water using dialysis tubing with 6-8k MWCO over 6 water changes. The water was removed by lyophilisation. The resulting polymers were redissolved in minimal amount of HPLC-grade 1,4-dioxane and sonicated before purification by prep-SEC in dioxane, collecting the reddish-purple band. The dioxane was removed by lyophilisation yielding the final pinkish-red **Zn-dPP-pDMA** (676 mg, 52 µmol, 78%).

1.4 Infra-Red (IR) Spectra

The IR spectra of the starting porphyrins (**3**, **3a** and **Zn-dPP**), polymers (**5** and **6**) and the final product **Zn-dPP-pDMA** are shown in Figure S3. In particular, the appearance of the terminal-alkyne stretch in **Zn-dPP** (blue dot) and azide stretch in **6** (orange dot) indicated the successful de-protection and azide functionalisation, respectively, of the starting compounds. Their subsequent disappearance demonstrated the successful coupling reaction.

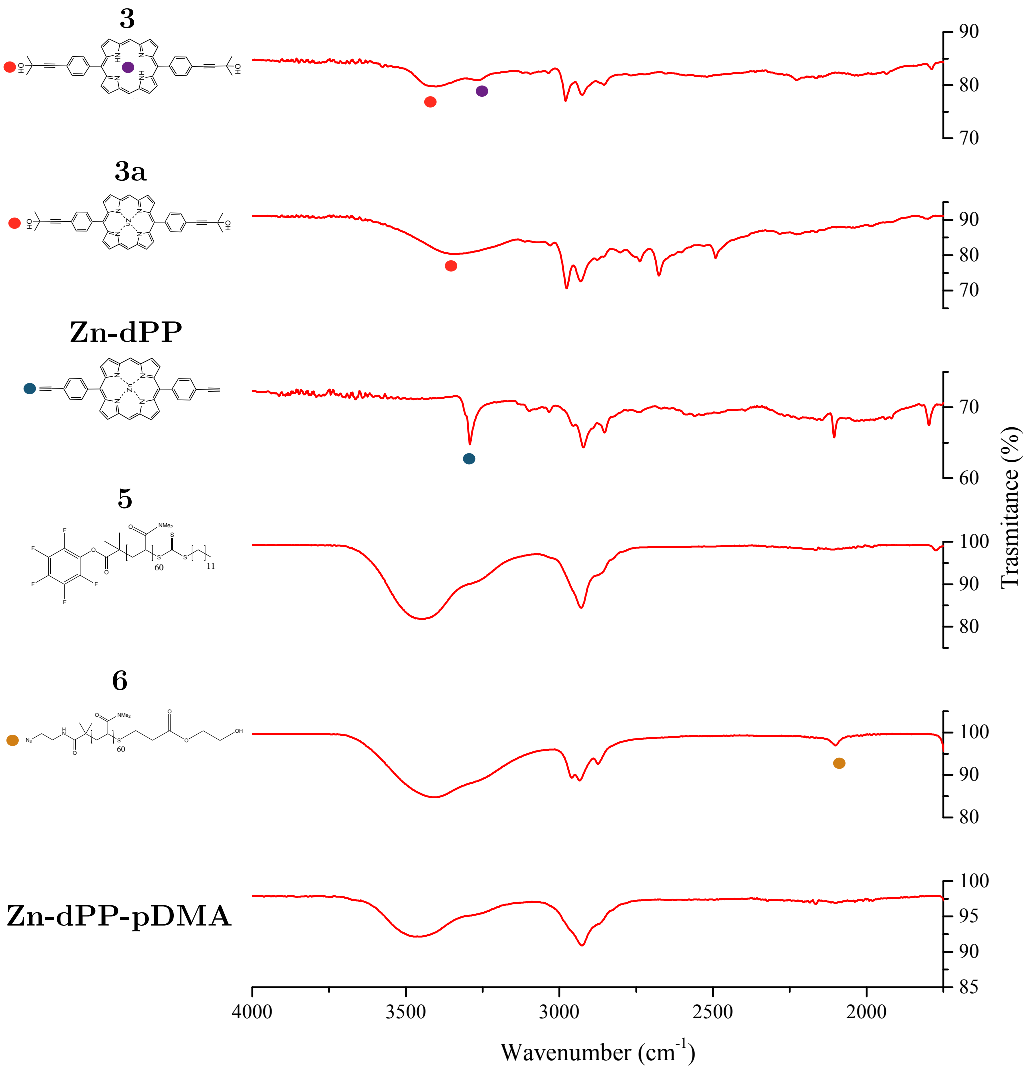

Figure S3 IR spectra of the starting and final products. Regions of interest are highlighted by dots of corresponding colours.

1.5 Size Exclusion Chromatography

The SEC spectra of the starting **pDMA** (RI) and **Zn-dPP-pDMA** (UV absorption at 414 nm) are shown in Figure S4. The anomalous shoulder at approximately double the molecular weight (Mw ≈ 28 kDa), which is attributed to the dimer of **Zn-dPP-pDMA**, is also observed, in accord with previously reported porphyrin-polymer conjugate systems.[9]

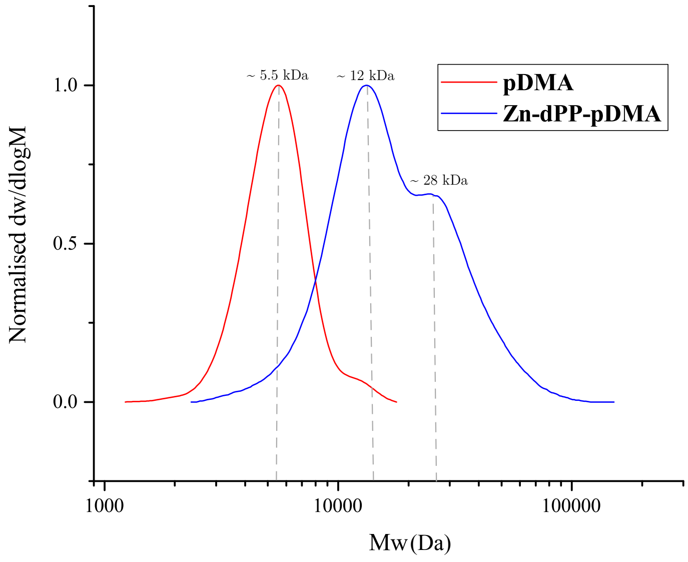

Figure S4 SEC of polymers in DMF with LiBr.

1.6 Assembly of Zn-dPP-pDMA

For a sample of 25 mL, 75 mg of **Zn-dPP-pDMA** was dissolved in 12.5 mL of dioxane and sonicated for 15-30 minutes. 12.5 mL of 18.2 MΩ·cm water was then added to the solution drop wise overnight *via* peristaltic pump. The resulting mixture was dialysed against 18.2 MΩ·cm water using dialysis tubing with 6-8k MWCO to slowly remove the dioxane over a minimum of 6 water changes. The solution was diluted with 18.2 MΩ·cm water when required for analysis.

2 Morphological Characterisation of Assembled Systems

2.1 Light Scattering

To determine the morphologies of the assembled system, light scattering measurements were performed. Initially, the results were inconsistent between each measurement and we were unsure whether the strong absorption of the **Zn-dPP** cores affected the readings. However, as the instrument uses 632 nm laser, it is outside the absorption range of **Zn-dPP**. We therefore examined our procedures more carefully and did measurements for different filtered as well as unfiltered systems (Figure S5). All samples were assembled as described in Section 1.5, followed by dilution from 3 mg/mL to 0.5 mg/mL prior to filtration and analysis. The radius of gyration (R_g) and hydrodynamic radius (R_h) as well as the R_g/R_h ratio of each measurements are summarised in Table S1.

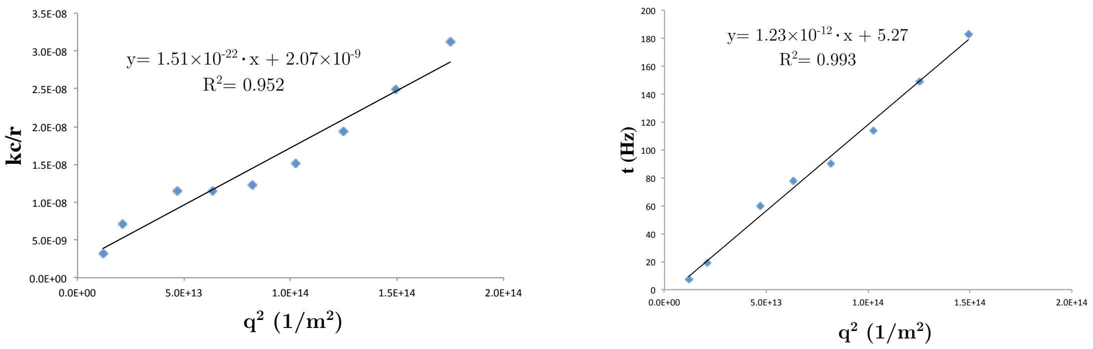

Figure S5 Representative static and dynamic light scattering data of unfiltered assembled systems.

Table S1 Fitted radii of filtrated assembled systems.

Filter pore-sizes	R_g/nm	R_h/nm	R_g/R_h
**220 nm**	55	34	1.6
**450 nm**	136	86	1.6
**Unfiltered**	420	190	2.4

As shown in Table S1, all radii of gyration (R_g) were approximately a quarter of the filter pore-sizes. This strongly suggested that the assembled systems underwent rearrangement upon filtration. The universal R_g/R_h of 1.6 suggested that the system under study were either elongated or of irregular formation

undergoing fusion/fission rearrangement.[10]

2.2 UV-Visible Absorption of Filtrated Samples

Since the unfiltered sample seemed to be multi-modal, we measured the UV-Visible spectra of the filtrated and unfiltered samples to investigate whether **Zn-dPP** were present in the large particles, shown in Figure S6. To our surprise, the filtration process seemed to have removed a rather large amount of **Zn-dPP** from the assembled systems. The Soret peak was reduced by *ca.* 40% and 50% post filtration, through 0.45 and 0.22 µm pores respectively. Although it is not unusual for samples to 'stick' to the filter membranes, removal of up to 50% of samples was 20 fold higher than the 2.5% observed decrease at the Q-band region in the test measurement with unimeric **Zn-dPP-pDMA** in dioxane at 6 mg/mL through the 0.22 µm filters (data not shown). This unusual loss of material indicated that **Zn-dPP** were indeed part of the large aggregates and were removed in the filtration process. However, the same slight red-shifts were present in all the spectra, suggesting that the filtration did not affect the micro-environment of the **Zn-dPP** core.

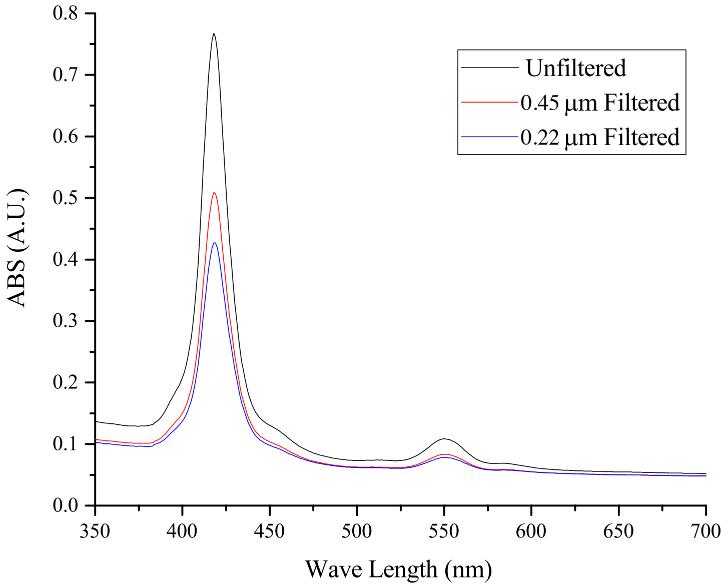

Figure S6 UV-Visible absorption spectra of filtrated samples. Samples were assembled in conditions detailed in Section 1.5 and diluted from 3 mg/mL to 0.5 mg/mL prior to filtration and measurements.

2.3 Transmission Electron Microscropy Studies

In addition to observing the unfiltered samples under cryo-TEM as shown in the main text, we also observed the filtered samples under dry state TEM. In agreement to our filtrated SLS/DLS studies, the size of the observed particles were roughly half the diameter of the filter pore-sizes. These are shown in Figures S7 and S8.

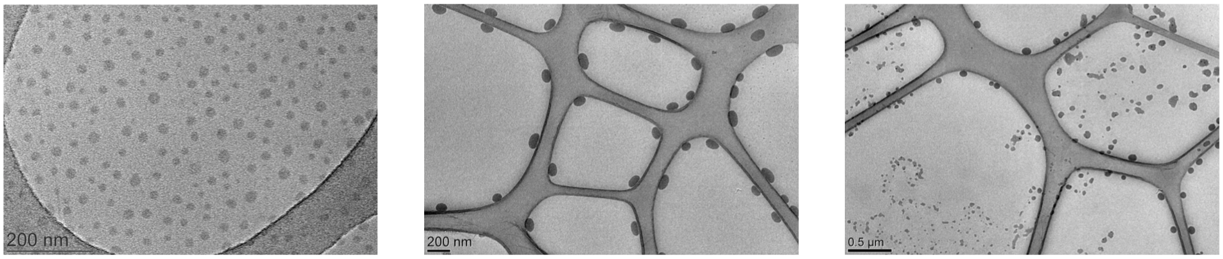

Figure S7 TEM images of **Zn-dPP-pDMA** assemblies filtered through 0.22 μm pores on GO coated girds.

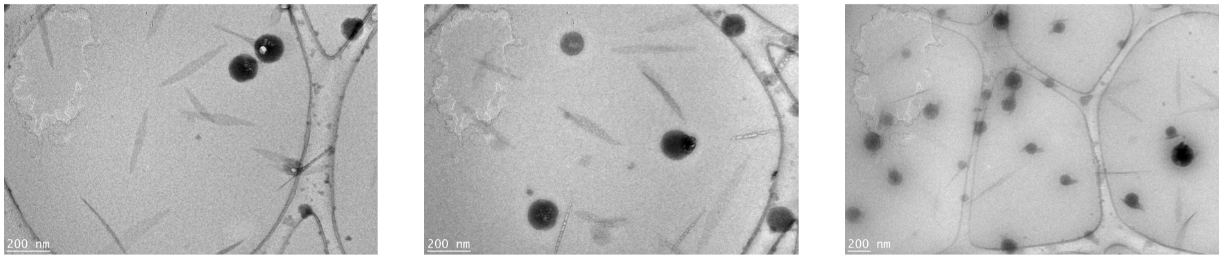

Figure S8 TEM images of **Zn-dPP-pDMA** assemblies filtered through 0.45 μm pores on GO coated grids.

As observed in these filtrated samples, even though some large spherical structures remained, the overall assemblies were damaged during the filtration processes, evidenced by the small circular fragments (0.22 μm filtered, Figure S7) and needle like structures (0.45 μm filtered, Figure S8). This, together with the SLS/DLS data (Section 2.1), strongly suggests that the assembled structures were extremely sensitive to shear forces applied during the filtration processes. Hence, in an effort to avoid both the loss of material and disturbance to their native assembled structures, all photochemical studies were performed with unfiltered samples.

3 Photochemical Information

3.1 Static Fluorescence

Heat maps for the measured fluorescence spectra of all systems are presented in Figure S9. All spectra were recorded with identical settings (excitation slit-width = 2.5 nm; emission slit-width = 5 nm; and photomultiplier tube voltage = 800 V). The spectra were uncorrected in energy, as the emission wavelength extends beyond 600 nm, which corresponds to the upper limit to which our instrument can correct for. Signals greater than the maximum fluorescence intensity (arising from instrument scattering) are all set to 0 for clarity. The emissions demonstrate similar features to those previously reported for Zn-*meso*-tetraphenyl-porphyrins.[11–14] In particular, modest emission following $S_2 \rightarrow S_0$ can be observed in both **Zn-dPP** and **Zn-dPP-pDMA** in dioxane; in the assembled system however, this feature appear to be relatively weakened, likely due to the increased scattering of the samples. Emission following $S_1 \rightarrow S_0$ showed the typical dual band features arising from the relaxation of S_1 into the two Franck-Condon active vibrational mode (FCAM) in the ground state: Q(0,0) centred at *ca.* 590 nm and Q(0,1) centred at *ca.* 640 nm, where the number of quanta of the dominant FCAM in the excited state and ground state respectively are given in parentheses. The fully solvated systems demonstrated almost identical ratio between the Q(0,0) and Q(0,1) peaks, similar to measurements in previous studies.[11–13] However, the ratio between these peaks were altered in the assembled system, the Q(0,0) showing stronger intensity relative to the Q(0,1) peak. This observation may indicate subtle differences in the geometry of the porphyrin in the S_1 and S_0 states, which lead to differences in the Franck-Condon factors. A more refined explanation of this difference would require vibrational frequency calculations that are likely to be prohibitively expensive in computational time and also beyond the scope of the present work.

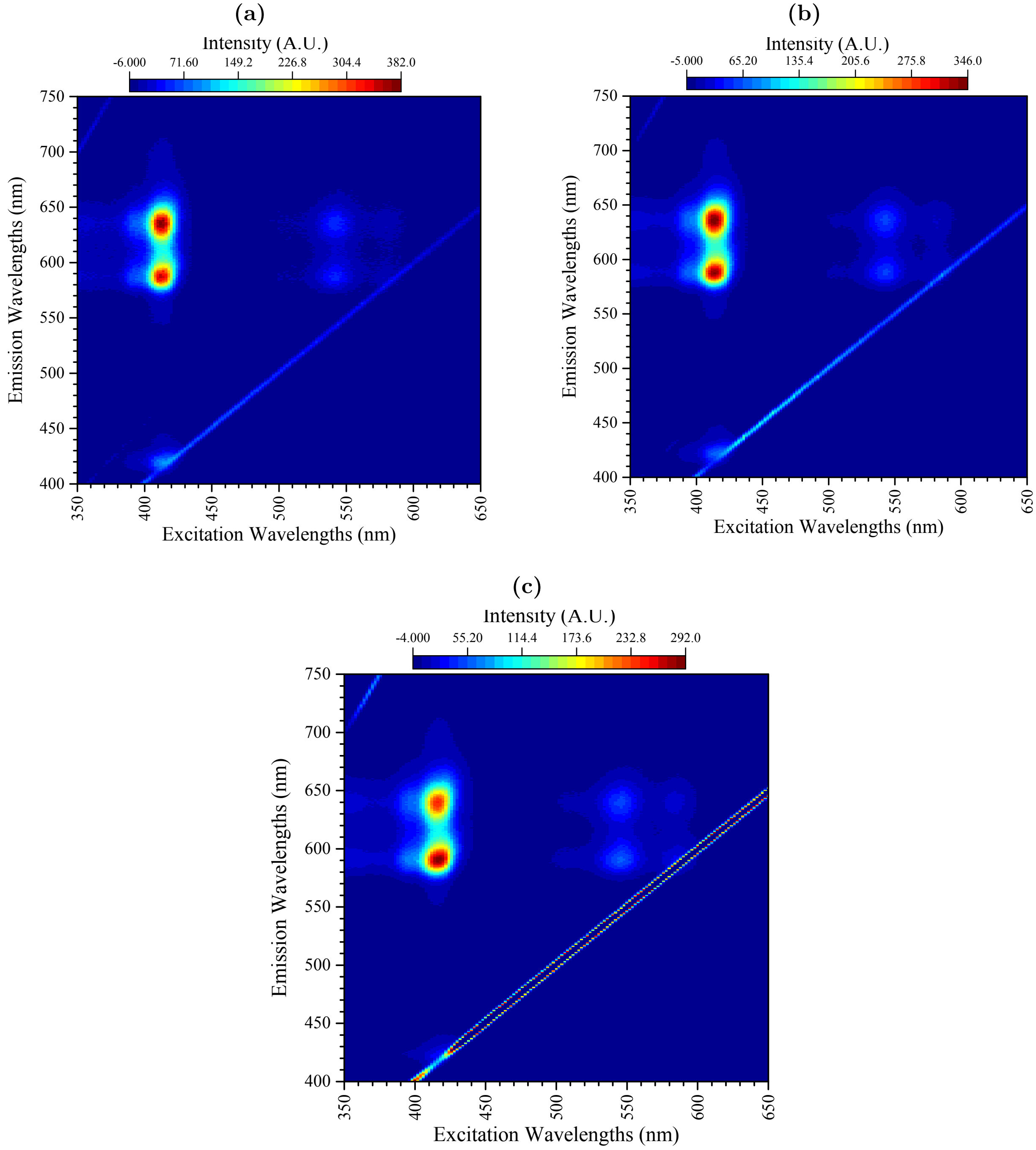

Figure S9 Static fluorescence heat map of (a), **Zn-dPP**, 57 μM in dioxane; (b), **Zn-dPP-pDMA**, 0.25 mg/mL (19.2 μM) in dioxane; and (c), **Zn-dPP** assembled as described in 1.4, diluted from 3 mg/mL to 0.33 mg/mL (25.6 μM) in 18.2 MΩ·cm water.

3.2 Time-resolved Transient Electronic Absorption Spectroscopy (TEAS)

Global Fitting and Error Analysis

A global fitting procedure is used to determine the set of lifetimes which characterise a function of four exponential decays convoluted with a Gaussian instrument response.[15] The full width at half maximum of the instrument response is measured to be $\sim$ 150 fs (determined through solvent only transients, data not shown). Representative fitted traces at 416 nm of the experimental data are shown in Figure S10.

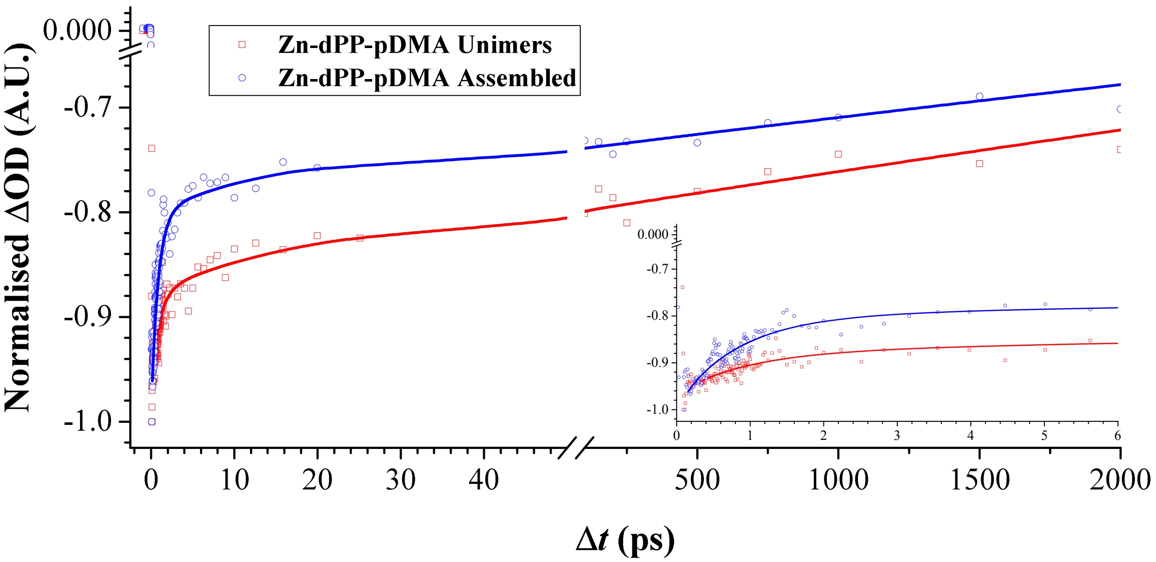

Figure S10 Normalised experimental data and the global fitted trace of **Zn-dPP-pDMA** both fully solvated in dioxane (**Unimers**) and assembled in water (**Assembled**), integrated at 416 nm.

We use support plane analysis to determine a 95% confidence interval on the lifetimes determined from global fitting.[16] One of these lifetimes is attributed to small spectral shifts in the TAS which is required for global fitting convergence. It is determined to be < 100 fs and therefore not considered as a resolvable dynamical process. Another lifetime behaves as a long-lived baseline offset returning a lifetime of $\gg$ 2 ns which we attribute to intersystem crossing ($\tau_{3/\mathrm{ISC}}$).[11] As a result, support plane analysis is only used for $\tau_{1/\mathrm{IC}}$ and $\tau_{2/\mathrm{IET}}$. Briefly, the goodness of fit, χ^2, for the globally fitted lifetimes is χ^2_{min}. The values of $\tau_{1/\mathrm{IC}}$ and $\tau_{2/\mathrm{IET}}$ are systematically varied, and for each pair of values, the fitting procedure reoptimises and returns a goodness of fit $\chi^2(\tau_{1/\mathrm{IC}}, \tau_{2/\mathrm{IET}})$. The ratio $\dfrac{\chi^2(\tau_{1/\mathrm{IC}}, \tau_{2/\mathrm{IET}})}{\chi^2_{min}}$ is calculated, and a 95% confidence

interval for the lifetimes is defined as:[17]

$$\frac{\chi^2(\tau_{1/\mathrm{IC}}, \tau_{2/\mathrm{IET}})}{\chi^2_{min}} = 1 + \frac{p}{\nu}\mathrm{F}^{-1}\left(0.95, p, \nu\right), \tag{1}$$

where p is the number of parameters used in the global fitting procedure, ν is the number of degrees of freedom and F^{-1} is the inverse-F cumulative distribution function . An upper bound on the uncertainty for the lifetimes is taken to be the value which satisfies the following two conditions; (i) is the largest deviation from the global lifetimes and (ii), satisfies equation 1.

Transient Absorption Spectra (TAS)

The TAS of the **Zn-dPP-pDMA** , both fully solvated and assembled are presented below. Almost identical features to the TAS of **Zn-dPP** are observed and are explained in detail in the main text.

(a)

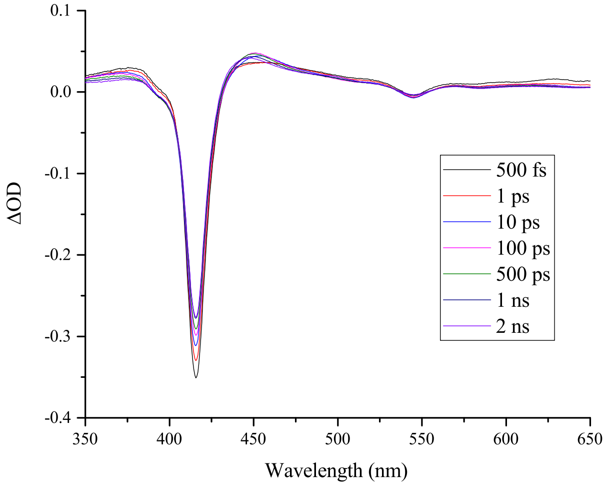

(b)

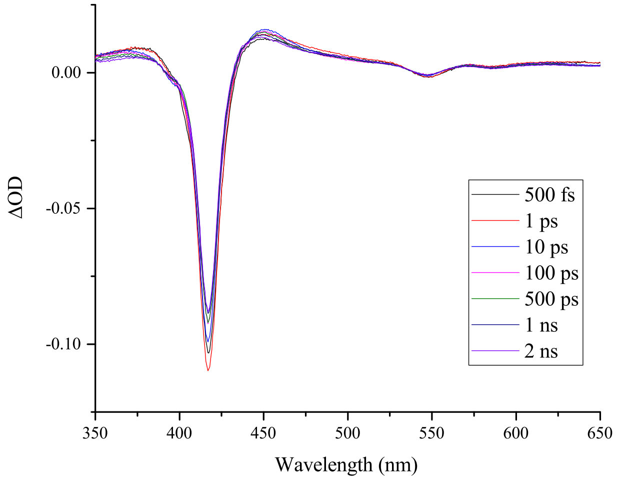

Figure S11 TAS of (a), **Zn-dPP-pDMA** (3 mg/mL, 250 μM) solvated in dioxane; and (b), **Zn-dPP** (3 mg/mL, 250 μM) assemebled in water. All TAS are recorded following excitation to S_2 state with 400 nm pump pulse.

Lifetime Uncertainties

Due to the dominant long time delay dynamics, the confidence level of $\tau_{2/\mathrm{IET}}$ extending to longer time delays is over estimated by our algorithm. We therefore quoted the error of $\tau_{2/\mathrm{IET}}$ as the distance from the origin to the furthest point towards $\tau_{1/\mathrm{IC}}$ in the main text (main text, Table 1). The 95% confidence level for each of the systems is highlighted by the bold black lines shown in Figure S12.

(a)

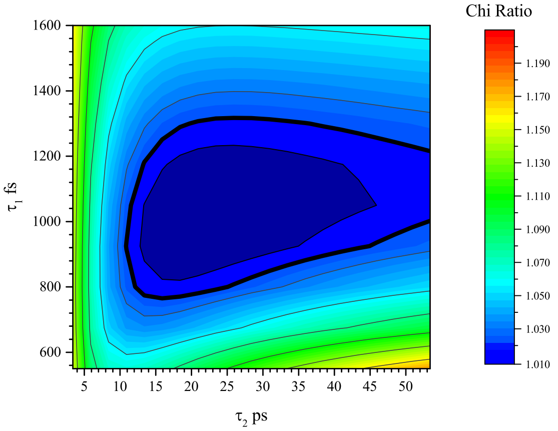

(b)

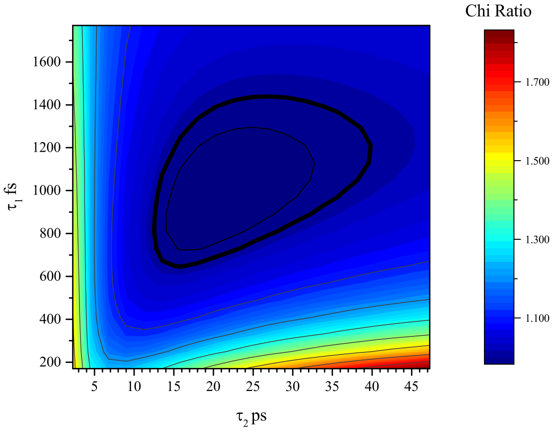

(c)

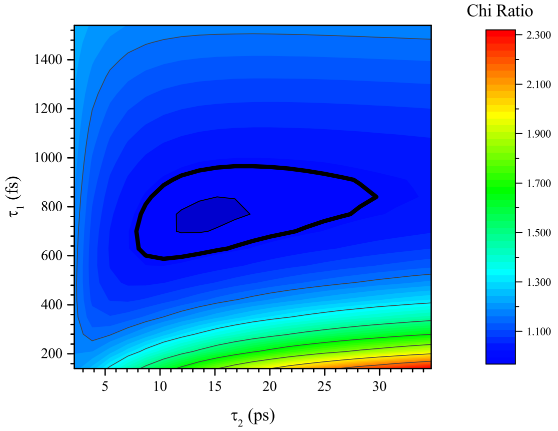

Figure S12 Chi ratio ($\chi^2(\tau_{1/\mathrm{IC}},\tau_{2/\mathrm{IET}})/\chi^2_{min}$) for $\tau_{1/\mathrm{IC}}$, $\tau_{2/\mathrm{IET}}$ of (a), **Zn-dPP** solvated in dioxane; (b), **Zn-dPP-pDMA** solvated in dioxane; and (c), **Zn-dPP-pDMA** assembled in 18.2 MΩ·cm.

References

[1] E. Stulz, S. M. Scott, Y.-F. Ng, A. D. Bond, S. J. Teat, S. L. Darling, N. Feeder and J. K. M. Sanders, *Inorg. Chem.*, 2003, **42**, 6564–6574.

[2] K. Godula, D. Rabuka, K. Nam and C. Bertozzi, *Angew. Chem. Int. Ed.*, 2009, **48**, 4973–4976.

[3] J. K. Laha, S. Dhanalekshmi, M. Taniguchi, A. Ambroise and J. S. Lindsey, *Org. Process Res. Dev.*, 2003, **7**, 799–812.

[4] T. R. Wilks, J. Bath, J. W. de Vries, J. E. Raymond, A. Herrmann, A. J. Turberfield and R. K. O'Reilly, *ACS Nano*, 2013, **7**, 8561–8572.

[5] J. P. Patterson, A. M. Sanchez, N. Petzetakis, T. P. Smart, T. H. Epps, III, I. Portman, N. R. Wilson and R. K. O'Reilly, *Soft Matter*, 2012, **8**, 3322–3328.

[6] S. E. Greenough, M. D. Horbury, J. O. F. Thompson, G. M. Roberts, T. N. V. Karsili, B. Marchetti, D. Townsend and V. G. Stavros, *Phys. Chem. Chem. Phys.*, 2014, **16**, 16187–16195.

[7] S. E. Greenough, G. M. Roberts, N. A. Smith, M. D. Horbury, R. G. McKinlay, J. M. Zurek, M. J. Paterson, P. J. Sadler and V. G. Stavros, *Phys. Chem. Chem. Phys.*, 2014, **16**, 19141–19155.

[8] M. Staniforth, J. D. Young, D. R. Cole, T. N. V. Karsili, M. N. R. Ashfold and V. G. Stavros, *J. Phys. Chem. A*, 2014, **118**, 10909–10918.

[9] D. A. Roberts, M. J. Crossley and S. Perrier, *Polym. Chem.*, 2014, **5**, 4016–4021.

[10] J. P. Patterson, M. P. Robin, C. Chassenieux, O. Colombani and R. K. O'Reilly, *Chem. Soc. Rev.*, 2014, **43**, 2412–2425.

[11] H.-Z. Yu, J. S. Baskin and A. H. Zewail, *J. Phys. Chem. A*, 2002, **106**, 9845–9854.

[12] P. G. Seybold and M. Gouterman, *J. Mol. Spectry.*, 1969, **31**, 1 – 13.

[13] D. J. Quimby and F. R. Longo, *J. Am. Chem. Soc.*, 1975, **97**, 5111–5117.

[14] R. Humphry-Baker and K. Kalyanasundaram, *J. Photochem.*, 1985, **31**, 105 – 112.

[15] A. S. Chatterley, C. W. West, V. G. Stavros and J. R. R. Verlet, *Chem. Sci.*, 2014, **5**, 3963–3975.

[16] L. A. Baker, M. D. Horbury, S. E. Greenough, P. M. Coulter, T. N. V. Karsili, G. M. Roberts, A. J. Orr-Ewing, M. N. R. Ashfold and V. G. Stavros, *J. Phys. Chem. Lett.*, 2015, **6**, 1363–1368.

[17] J. R. Lakowicz, *Principles of Fluorescence Spectroscopy*, Springer Science+Business Media, 3rd edn, 2006.
